# Identifying sources of metabolomic diversity and reconfiguration in peach fruit: taking notes for quality fruit improvement

**DOI:** 10.1002/2211-5463.13233

**Published:** 2021-07-20

**Authors:** María F. Drincovich

**Affiliations:** ^1^ Centro de Estudios Fotosintéticos y Bioquímicos Consejo Nacional de Investigaciones Científicas y Técnicas Facultad de Ciencias Bioquímicas y Farmacéuticas Universidad Nacional de Rosario Argentina

**Keywords:** fruit, metabolic diversity, metabolomic, peach, quality, reconfiguration

## Abstract

The metabolomic content determines many of the important features of a fruit, such as its taste, flavor, color, nutritional value, and abiotic or biotic resistance. Peach (*Prunus persica* (L.) Batsch) is one of the best genetically characterized species used as a model for Rosaceae, the drupes of which are a source of minerals, vitamins, fiber, and antioxidant compounds for healthy diets around the world. During the last few years, a great advance in the analysis of the metabolic diversity and reconfiguration in different peach varieties in response to developmental and environmental factors has occurred. These studies have shown that the great phenotypic diversity among different peach varieties is correlated with differential metabolomic content. Besides, the fruit metabolome of each peach variety is not static; on the contrary, it is drastically configured in response to both developmental and environmental signals, and moreover, it was found that these metabolic reconfigurations are also variety dependent. In the present review, the main sources of metabolic diversity and conditions that induce modifications in the peach fruit metabolome are summarized. It is postulated that comparison of the metabolic reconfigurations that take place among the fruits from different varieties may help us better understand peach fruit metabolism and their key drivers, which in turn may aid in the future design of high‐quality peach fruits.

## Graphical abstract

Fruit quality is closely related to its metabolic composition, which defines taste, flavor, color, nutraceutical properties, and resistance degree to environmental conditions. Chemical composition of peach fruit is highly diverse: The different peach varieties display differential metabolomic content, which is further highly modulated by post‐ and preharvest management and development. Knowledge about this chemical diversity is key for peach quality improvement.

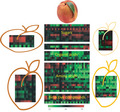

**Abbreviation**
CI: chilling injury

Plants, as sessile organisms, produce large amounts and diverse types of metabolites to respond to the widely different array of environments in which they must adapt to live. Metabolites are the products of transcriptomic and proteomic changes in response to developmental and environmental signals, and protect plants against abiotic stresses, such as drought, high light, photoinhibition and UV irradiance, high and low temperatures, and toxic metals. Metabolites also constitute effective defenses against biotic stresses and are part of acquired defense system as well. Thus, the metabolic content of a cell has been described as bridging the genotype–phenotype gap [1], because it reflects transcriptomic and proteomic changes linked to genetic and epigenetic regulation. Furthermore, the metabolic levels of a cell play a strategic role in signaling, interfacing with various molecular processes, and modulating transcriptomic and proteomic changes [2, 3] (Fig. 1). Since their initial applications [4], nontargeted and comprehensive metabolomic profiling studies of different plant organs, tissues, and/or cells have proven to be key in assessing genotypic and phenotypic diversity, in defining biochemical changes associated with development processes or in response to environmental signals, in comparative compositional studies, and in food quality assessments [5].

**Fig. 1:**
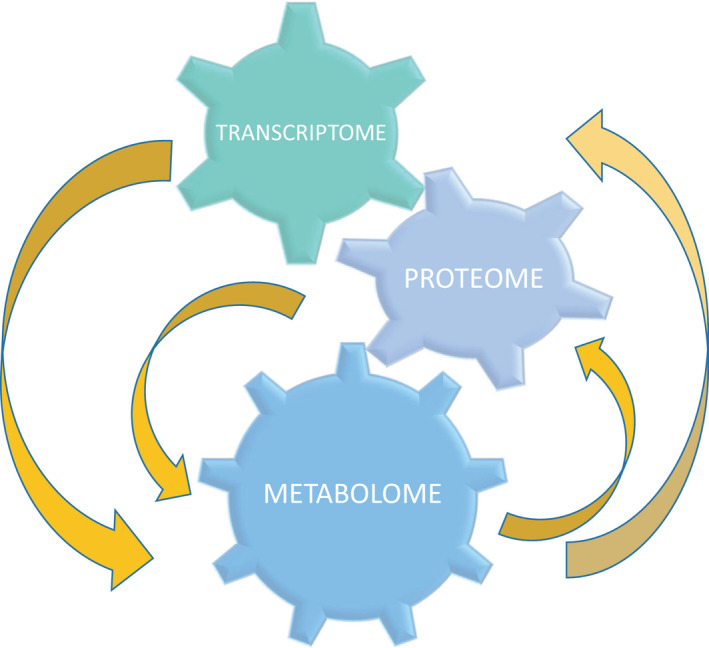
The figure shows that the metabolome is not only the end product of transcriptomic and proteomic changes, but also plays a strategic role in signaling, interfacing with various molecular processes, and modulating transcriptomic and proteomic changes.

Among the different organs of the plants, fleshy fruits display a highly diverse metabolic content, in accordance with its role of attracting fruit‐eating organisms for seed dispersal. Besides, during fruit development, several different metabolites accumulate to discourage consumption, while waiting for the seed to be mature. In this sense, the fruit is one of the most metabolite‐rich organs of the plants and as such contains a large range of chemical complements: metabolites involved in taste and flavor; those with nutraceutical properties; and those with defense properties against biotic and abiotic stress. Moreover, the overall fruit quality traits are closely related to their metabolic composition [6]. A broad range of fruit phenotypic variations can be found in angiosperms [7]. Among the different types of fleshy fruits, drupes are present across different plant families, including economically important crops such as peach, plum, cherry, almond, coffee, raspberry, and others. Many species belonging to Rosaceae have this kind of fruit, which is characterized by a lignified endocarp that protects the seed [8, 9]. Peach (*Prunus persica*) is one of the best genetically characterized deciduous trees used as a model for Rosaceae [8], and the drupes of this species have a fleshy mesocarp which are a source of minerals, vitamins, fiber, and antioxidant compounds for healthy diets around the world [10]. In recent years, untargeted metabolomic approaches have been used to analyze the metabolic content of peach fruits from different varieties and subjected to different pre‐ and postharvest conditions; such studies revealed great metabolic diversity in this fruit. As overall peach quality depends on its metabolomic content, the main sources of metabolic diversity and conditions that induce modifications in the peach fruit metabolome are summarized in this review.

## Sources of metabolic diversity in peach

### Peach cultivars: a general source of fruit metabolic diversity

Cultivated peach shows a wide degree of variation in their fruit traits, with great differences in fruit size, texture, flavor, sweetness/acidity ratios, and/or skin and flesh color. The different cultivated peach varieties were developed in different countries to satisfy diverse demands, such as higher yield, expansion to different production zones, disease resistance, and superior postharvest quality [11]. Genome analysis of several different cultivated peach and closely related relatives suggests that genes related to an increase in fruit size, skin color, and those related to increased sugar content were selected during peach domestication and improvement [12, 13, 14, 15, 16].

Fruit of modern cultivated peach varieties belong to *P. persica* and show different phenotypic properties [11, 17]. According to their fruit texture and firmness, peach cultivars are classified as melting flesh, showing soft and juicy fruit when fully ripe; nonmelting flesh, and stony hard fruits, which remain firm after harvest [18]. Depending on the time of harvest in the year, peach varieties are classified as early, mid, or late harvesting date. Flesh adhesion can also be used to divide peach cultivars into freestone, semi–freestone, or clingstone. Several different epidermal or flesh pigmentations, such as yellow, white, and red, can be also found among the fruits of the different peach cultivars. Nectarines display no pubescence on the fruit surface, a characteristic that is controlled by a single or few linked genes [19]. Depending on the fruit acidity, peach fruits are further classified into high‐acid and low‐acid cultivars. Thus, the wide diversity of peach cultivars that were developed, and are still being developed, in different peach breeding programs around the world, offers consumers a great array of taste, texture, and flavor possibilities, with different nutritional and health benefit properties. I should mention the peach reference collection recently developed across different European countries, which all share the same experimental design to conserve and explore peach germplasm resources [17].

In the last few years, nontargeted metabolomic studies of the fruit of different peach cultivars have shown that the great phenotypic diversity is correlated with highly differential metabolomic content. The fruit from the different cultivars shows great diversity in the levels of sugars, organic acids, amino acids, lipids, and volatile compounds [20, 21, 22]. Moreover, targeted evaluation of the levels of sugars, organic, and amino acids in the fruit from different peach cultivars has revealed wide variation of these key carbon compounds involved in fruit taste and quality [23, 24, 25, 26, 27, 28]. The type and content of volatile compounds, which contribute to fruit aroma and flavor, are also variety dependent [29]. The level of several bioactive compounds, minerals, vitamins, flavonoids, phenolic composition, antioxidant capacities, and metal chelating activity is also dependent on the peach variety studied [28, 30, 31, 32, 33, 34]. Analysis of the population structure of several different peach accessions indicated that genes associated with high polyphenol composition decreased during peach domestication and improvement, indicating breeding potential for obtaining peach fruit with enhanced bioactive polyphenols [16].

### Metabolomic changes during fruit development and ripening

**Fig. 2:**
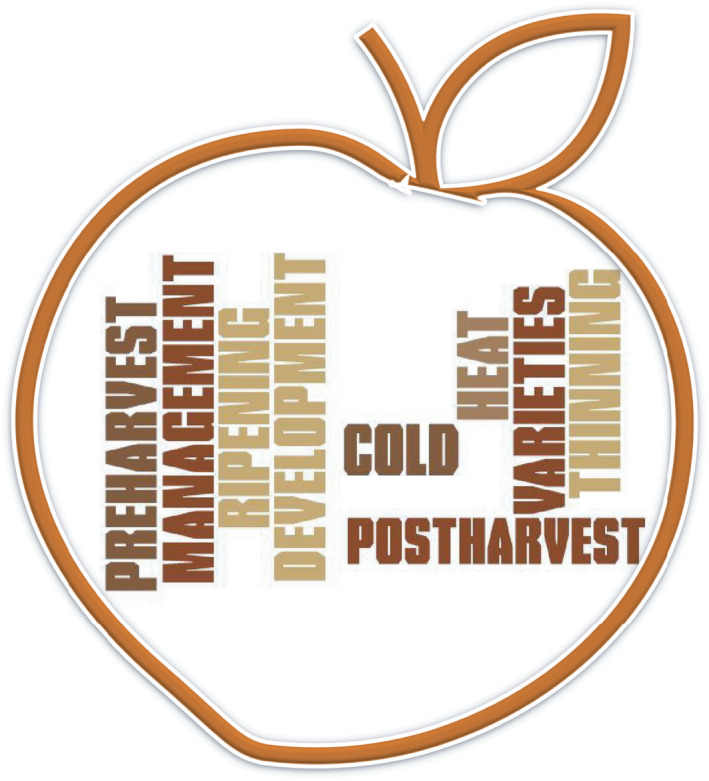
The main sources of metabolomic diversity and conditions that induce metabolic reconfigurations in peach fruit, as discussed in the present review.

Besides the high level of variation in the fruit metabolome when comparing different peach varieties, the fruit metabolome of each peach variety is not static; on the contrary, it is drastically configured in response to both developmental and environmental signals during both pre‐ and postharvest managements (Fig. 2).

The development and ripening of fruits are dynamic processes that comprise complex molecular and biochemical changes, which are linked to great metabolome modifications. Peach development is divided into well distinct stages (S1 to S4) [35]. The first stage after fruit set (S1), characterized by a rapid increase in cell division and elongation, is followed by the second stage (S2), which is characterized by endocarp lignification to form the stone, with practically no increase in fruit size. During the third stage (S3), a rapid increase in fruit size takes place concomitant with rapid cell division, while in the final stage (S4), the peach fruit reaches the final full size. Nontargeted metabolomic studies during development of a particular peach variety have shown that each stage of development is characterized by specific metabolic programs [36]. Moreover, differential biochemical processes take place in distinct tissues during early peach development, such as lignin biosynthesis in the endocarp and flavonoid biosynthesis in the mesocarp and exocarp [37, 38]. The study of sugar metabolism during fruit development in a peach progeny of genotypes with contrasting fructose to glucose ratios indicated differences in the time course accumulation of the sugars analyzed, as well as a poor correlation between enzymes involved in sugar metabolism and metabolite concentrations [39]. The study of the dynamic changes in organic acid accumulation in the fruit of different peach cultivars throughout development revealed different patterns of organic acid accumulation in high‐ and low‐acid cultivars [26].

Peach is a climacteric fruit that undergoes ripening after reaching the maturation stage at S4. The drastic physiological and biochemical changes that take place at this stage transform the fruit into an edible and attractive organ. The comparison of the ripening process across different peach varieties by nontargeted metabolomic approaches revealed both conserved and distinct metabolic processes among varieties associated with this complex process [20]. The levels of bioactive compounds, such as polyphenols, flavonoids, and condensed tannins, at different ripening stages also varied among different peach varieties [31, 34]. Thus, in addition to the complex metabolic modifications that take place in each peach fruit variety during development and ripening, particular metabolic programs associated with these processes can be found among fruits from diverse peach varieties.

### Metabolomic reconfigurations during postharvest management and storage

Peaches deteriorate quickly after harvest, so they are stored at low temperature to extend their commercial and shelf life. Cold storage delays ripening and softening and reduces enzymatic and microbial activity; and ripening is restored when the fruit is returned to ambient temperature. Apart than being essential for commercialization, cold storage induces in the fruit a complex response program, resulting in a global reconfiguration of the metabolome, which impacts on the subsequent ripening process. Untargeted metabolomic approaches in several different peach varieties were used to analyze the metabolome reconfiguration of the fruit by postharvest cold storage [22, 40, 41, 42]. Comparison of cold‐induced metabolome reconfiguration among different peach varieties showed that the metabolic modifications are dependent on the variety, the extent of cold treatment, and the temperature of storage [22, 40, 42]. Moreover, cold storage induces several different changes in the peach lipidome, which were also dependent on both the extent of cold storage and the peach variety [21].

When the different molecular modifications induced by cold are unsuccessful at protecting the fruit against cold, low temperature produces fruit damage and the development of the disorder called chilling injury (CI). CI includes mealiness, browning, loss of flavor or loss of the ability to ripen, and increased decay incidence in peach. Tolerance to CI is a multigenic trait in peach: It depends on a complex molecular rearrangement to build up a proper molecular defense against cold. Several different postharvest treatments have been successfully applied to peach fruit to ameliorate CI symptoms, such as the application of several different physical (as temperature) treatments and/or chemical compounds, such as salicylic acid, methyl jasmonate, aminobutyric acid, or gibberellic acid, among others. In this sense, preconditioned treatments (48 h at 20 °C or 8 °C for 5 days) before cold storage were used to decrease CI symptoms. These treatments modified the metabolome in the genotypes in which they were studied [43, 44]. In other studies, heat treatment prior to cold treatment [41] induced a metabolome modification that was able to prime the fruit to cope with cold storage. It remains to be established if the modifications induced by these pre cold treatments are differential or not when comparing different peach genotypes. The comparison of peach fruit of the same variety with contrasting woolliness phenotype [45] or among siblings with contrasting juice content [46] showed significant differences in the levels of metabolites. Overall, it seems that differences in postharvest management can modify a diverse array of biochemical components to cope with cold stress to alleviate CI symptoms, processes that are also highly dependent on the genotype.

### Differences in preharvest management impact on the metabolic content of fruit

Preharvest factors have a great impact on the final quality of peach fruit [47]. Among these preharvest factors, modifications of competition for resources among fruits on the same tree, the position of fruits on the tree, differential light exposure in the tree due to canopy management, among others, can affect sink strength and, therefore, are expected to affect the metabolome content of each fruit [48]. Nontargeted metabolite profiling was recently used for comparison of peach fruits at different developmental stages from unthinned or thinned peach trees [49, 50]. Thinning is a widespread management that balances the source–sink relationship reducing the competition among fruits. High carbon supply during early fruit development, as occurs in thinned peach trees in comparison with unthinned, can significantly affect fruit quality at a mature stage. Furthermore, the levels of some specific metabolites at early developmental stages correlate with the final quality of the fruits in the two varieties studied [49]. These studies clearly indicate that preharvest management has a great impact on the final metabolome and quality of peach fruits and that levels of some metabolites at early developmental stages correlate with the final fruit quality. It remains to be established if metabolites that have the potential to predict the final peach fruit quality are common or distinct when comparing different peach varieties.

## Metabolic diversity inside the fruit: the importance of profiling different tissues and cells

The different plant organs, tissues, and cellular and subcellular compartments are characterized by a specific metabolite composition and metabolism. Although it is often difficult to isolate specific cell types or subcellular compartments, this level of complexity should also be taken into consideration when studying the metabolism of peach fruit. The peach fruit is a complex organ, in which the exocarp envelops the edible and fleshy mesocarp, which in turn surrounds the endocarp that hardens during development and encloses the seed.

Some studies have separated the endocarp, mesocarp, and exocarp parts during development of the fruit, showing the coordination and competition of different metabolic processes among these tissues [37, 38, 51]. On the other hand, although the majority of the metabolomic studies performed in mature peach fruit have focused on the mesocarp, which is the edible part of the fruit, some studies have separately analyzed the metabolic content of the mesocarp and exocarp of fruits from several different peach varieties [28, 30, 32]. These studies have shown that these different fruit tissues display differential metabolic and bioactive compounds, the contents of which are also dependent on the variety. In particular, the exocarp of fruits shows high antioxidant capacities and content of bioactive compounds, suggesting that this part of the peach fruit can be used for obtaining health‐promoting compounds.

## Conclusions

The metabolomic content determines many of the important features of a fruit, such as its taste, flavor, color, nutritional value, abiotic stress, and disease resistance, among others. The fruit metabolome is dynamic, as it is constantly changing to respond to several different developmental and environmental signals and is also able to modulate transcriptomic and proteomic changes. During the last few years, a great advance in the analysis of metabolic diversity and reconfiguration in different peach varieties in response to developmental and environmental factors has occurred. Comparison of the metabolic changes among fruits from different varieties is a valuable way of enhancing our understanding of peach fruit metabolism, which could help in the identification of the key drivers of primary metabolism regulation, and thus, of fruit growth and quality. A future challenge is the integration of metabolomic information with transcriptomic and proteomic data in peach fruit, to match the level of key metabolites to gene clusters. This integration, along with the identification of common and differential metabolic processes among distinct peach varieties, will help in the design of superior and more nutritious fruit to feed a growing world population.

## Conflict of interest

The author declares no conflict of interest.
